# Case report: A rare occurrence of a huge mediastinal mass from disseminated prostate carcinoma

**DOI:** 10.1016/j.amsu.2021.102952

**Published:** 2021-10-14

**Authors:** Kai Ming Teah, Ching Pei Bong, Kamarudin Mudin, May Zaw Soe, Shankaran Thevarajah, Boon Tat Yeap

**Affiliations:** a Department of Anaesthesiology and Intensive Care, Faculty of Medicine and Health Sciences, Universiti Malaysia Sabah, 88400, Kota Kinabalu, Sabah, Malaysia; b Department of Orthopaedics, Queen Elizabeth Hospital 2, 88300, Kota Kinabalu, Sabah, Malaysia; c Department of Medical Education, Faculty of Medicine and Health Sciences, Universiti Malaysia Sabah, 88400, Kota Kinabalu, Sabah, Malaysia; d Department of Urology, Queen Elizabeth Hospital 2, 88300, Kota Kinabalu, Sabah, Malaysia

**Keywords:** Mediastinal mass, Prostate carcinoma, Disseminated, Anaesthesia, Prone position

## Abstract

**Background:**

Prostatic carcinoma is the commonest malignancy among men. It usually metastasizes to the spine and regional lymph nodes. However, it is extremely rare for it to metastasize to the mediastinum.

**Case presentation:**

An elderly man presented to us with progressive onset of bilateral lower limb weakness which was associated with thoracic radiculopathy and urinary incontinence. An urgent magnetic resonance imaging (MRI) of the spine showed severe cord compression with enlarged prostate and superior mediastinal mass. A computed tomography (CT) guided biopsy of the mediastinal mass was suggestive of prostatic malignancy. An emergency posterior instrumentation and fusion (PSIF) in prone position was successfully done. Histopathological examination of the spine showed malignant glandular tissues, suggestive of prostate.

**Discussion:**

A huge mediastinal mass can compromise the cardiorespiratory system and is very challenging for the anaesthetist to safely secure the airway for surgical procedures. Androgen deprivation therapy (ADT) for patients with metastatic prostatic carcinoma can be achieved either by medical castration or with bilateral orchidectomy.

**Conclusion:**

It is extremely uncommon for a prostatic carcinoma to metastasize to the mediastinum. Patients with a huge mediastinal mass possess risks of cardiorespiratory collapse perioperatively. Chemoradiotherapy and androgen deprivation therapy (ADT) can be utilized for metastatic prostatic carcinoma with good outcomes.

## Highlights

• Distant metastasis to the mediastinum is extremely rare.
• Patients with a huge mediastinal mass are at risks of cardiorespiratory collapse.
• Anaesthesia for patients with a huge mediastinal mass in prone position carries significant risks.
• Androgen deprivation therapy (ADT) for patients with metastatic prostatic carcinoma can be achieved either by medical or surgical castration.

## 1 Introduction

Prostatic carcinoma is the most prevalent malignant disease among men worldwide [1]. It usually metastasizes to the spine via the hematogenous and lymphatic routes. Other sites of distant metastasis including the mediastinum are extremely uncommon. We herein report a case of successful anaesthetic management of a patient diagnosed with disseminated prostate carcinoma with bone and mediastinal metastasis who underwent emergency spine surgery in prone position. This work has been reported in line with the SCARE criteria [2].

## 2 Case presentation

A 68-year-old healthy male (weight = 60kg and height = 172cm) presented with deteriorating bilateral lower limb weakness and numbness over a five-day period, which was associated with thoracic radiculopathy radiating along his abdomen. The patient did not complain of back pain and had no history of physical injury. He also did not exhibit weight loss and lack of appetite. He reported experiencing occasional urinary incontinence, frequency, intermittency, nocturia and terminal dribbling with inability to control flatulence over the past one year. However, he did not seek any medical treatment due to the long distance between his home and the hospital. The patient did not admit to having fever, shortness of breath, stridor, close contact with patients with pulmonary tuberculosis (PTB), and any family history of malignancy.

Upon examination, he was found to exhibit spine tenderness at the lower thoracic region, with paraplegia and absence of sensation at the level of tenth thoracic vertebra (T10) and below, which corresponded to complete spinal cord injury with American Spinal Injury Association (ASIA) A neurology. However, his prostate was enlarged with an estimated size of 5cm × 3cm, hard in consistency and with its surface irregular. Other clinical examinations were insignificant.

**Fig. 1:**
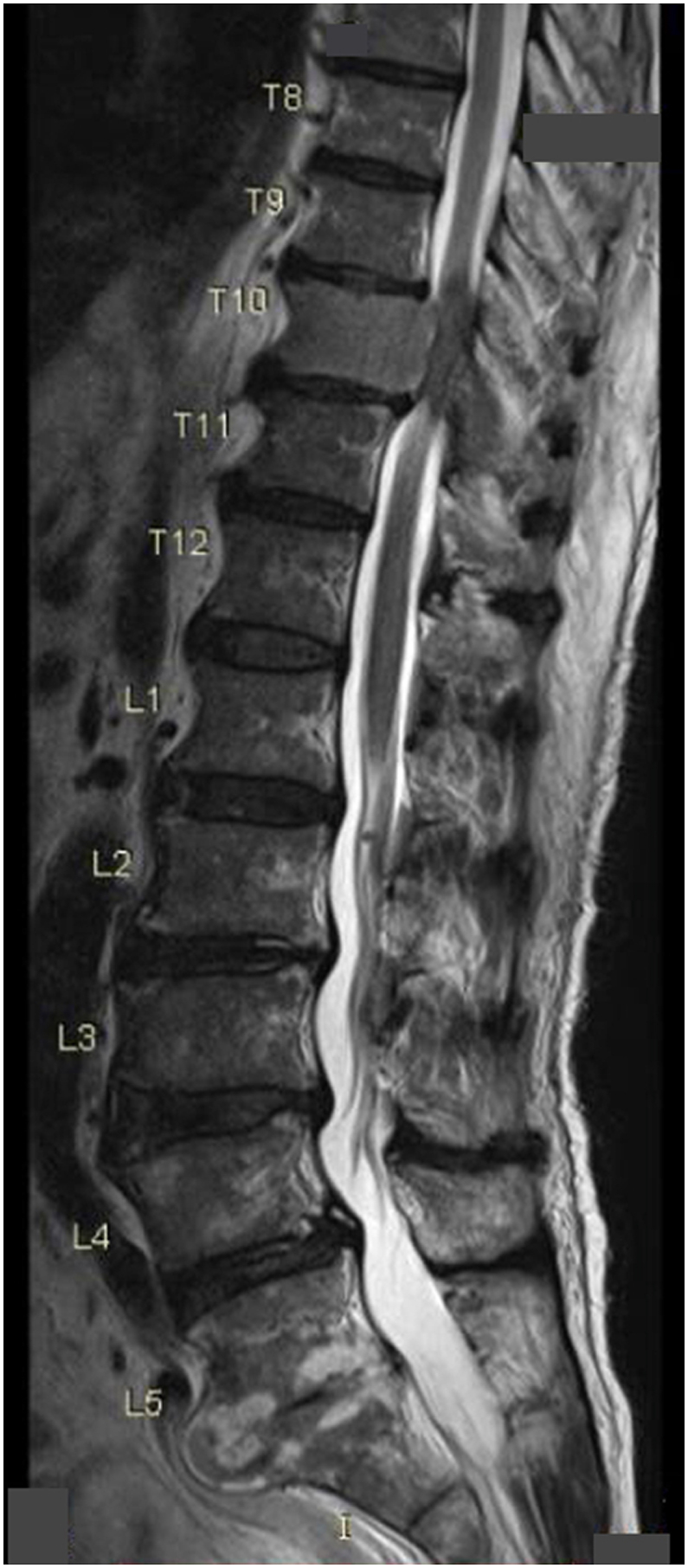
Sagittal view of the spine MRI showing severe spinal cord stenosis at T10 level.

**Fig. 2:**
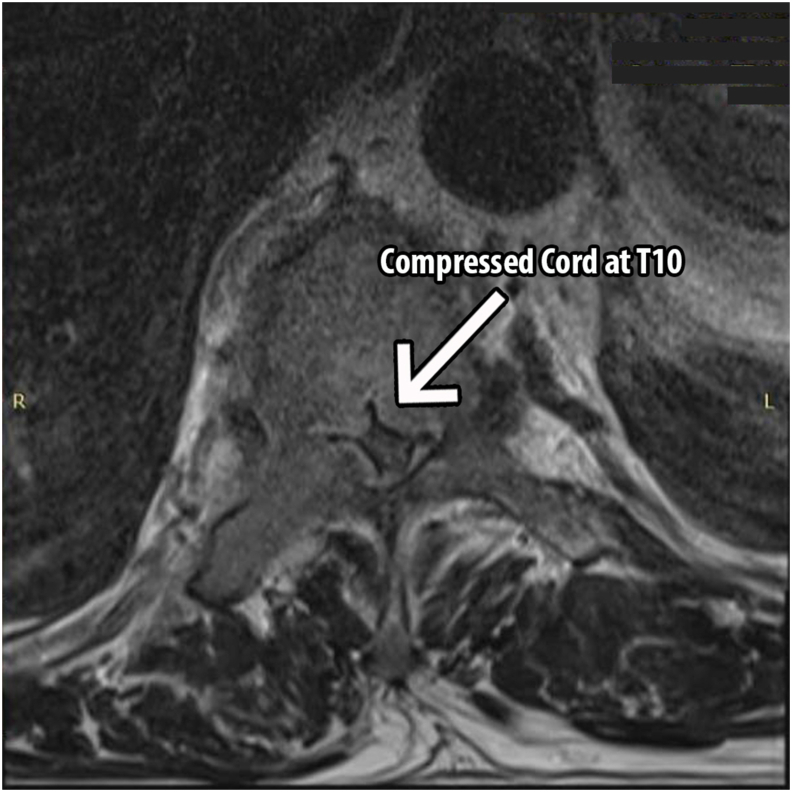
Axial view of the spine MRI.

**Fig. 3:**
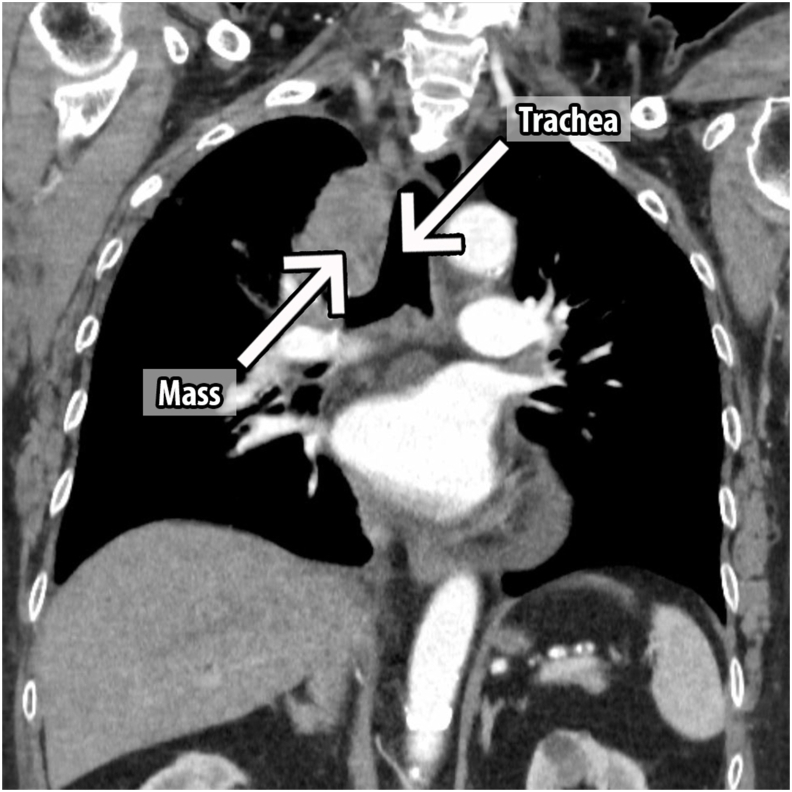
CT TAP showing a huge mediastinal mass.

An urgent magnetic resonance imaging (MRI) of the spine showed severe cord compression at the T10 level and a large right-side anterior mediastinal mass measuring 7.5cm × 4.3cm x 7.5cm which extended laterally to the right sternoclavicular joint and inferiorly to the right atrium. However, it did not cause any gross impingement to the large airways and vessels [Fig. 1, Fig. 2]. A computed tomography (CT) guided biopsy of the mediastinal mass under local anaesthesia showed metastatic carcinoma which was most likely prostate in origin. The immunohistochemical (IHC) staining was positive for prostate specific antigen (PSA) and AR (androgen receptor). There were also dispersed individual ill differentiated atypical large glandular cells that did not form clusters and were set in a dense background of immature lymphoid cells. The former showed large vesicular nuclei with conspicuous nucleoli and moderate nuclear polymorphism whereas the dense immature lymphoid cells were medium-sized mononuclear blue cells with high nucleo-cytoplasmic ratio, hyperchromatic nuclei and fine chromatin. A contrasted CT of the thorax, abdomen and pelvis (CT TAP) revealed a significantly enlarged prostate in addition to the large right anterior mediastinal mass. No abnormalities were detected in other organs [Fig. 3]. Serum prostate specific antigen (PSA) was grossly elevated at 293ng/ml (normal values: <4 ng/ml). Other tumour markers namely carcinoembryonic antigen (CEA) and carbohydrate antigen 19-9 (CA 19-9) were within normal range. His serum acetylcholine receptor antibodies were negative thus ruling out thymoma and Myasthenia Gravis in the patient. Other endocrine, biochemical and haematological parameters were within normal values.

The spine surgical team decided on an emergency posterior spinal instrumentation and fusion (PSIF) of the levels from T8 to T12 in prone position under general anaesthesia (GA) and guided by motor evoked potentials (MEP) and somatosensory evoked potentials (SSEP) monitoring. The anaesthetic team secured the patient's airway by awake fibreoptic intubation (AFOI) using the Spray-As-You-Go (SAYGO) method supplemented with remifentanil targeted controlled infusion (TCI) as sedation. AFOI was successfully performed with a size 7.5mm flexometallic endotracheal tube (FM ETT). GA was maintained with propofol TCI at the range of 3–5.5 mcg/ml and remifentanil TCI at 2–7 ng/ml, without the usage of muscle relaxants. The right radial artery and right femoral vein were cannulated for blood pressure monitoring and central venous access. The patient's mean airway pressure (MAP) was 21–24 cmH20 and tidal volume (TV) 400–450ml in supine position. After prone positioning, the MAP and TV remained relatively the same.

Intraoperatively, there were no episodes of haemodynamic instability or desaturation. The T10 tumour was removed via transpedicular piecemeal vertebrectomy to release the cord compression. The spine was subsequently stabilised with posterior instrumentation from T8 to T12 guided by SSEP and MEP. An estimated blood loss of 1.5L was adequately replaced with a mixture of crystalloids and colloids.

Postoperatively, the patient was extubated without any complications. Analgesia was maintained using patient-controlled analgesia (PCA) with morphine and intravenous paracetamol. His lower limbs showed improved motor and sensory functions from ASIA A neurology to incomplete cord injury with ASIA C neurology.

The HPE findings of the spine showed fragments of bony trabeculae with marrow admixed with fatty tissues. Foci of ill differentiated cribriform malignant glandular cells, suggestive of prostate glands infiltration, were observed within the marrow. The cells were enlarged and pleomorphic with prominent nucleoli and occasional mitosis. The malignant cells were immunoreactive towards CK AE1/AE 3, AR and PSA while negative towards CK 7 and CK 20 which confirmed the diagnosis of spine metastasis from prostate.

Since his discharge from the hospital, the patient underwent follow-up sessions at the combined oncology clinic with a good compliance to regular physiotherapy and rehabilitation. He was prescribed lifelong subcutaneous goserelein acetate (Zoladex), a luteinising hormone releasing hormone (LHRH) agonist, administered once every 3 months to control progression of prostate carcinoma. He commenced chemoradiotherapy after a month and had regular follow-up. The patient was able to move both lower limbs with support and to urinate with a good flow of urine. A surveillance scan 6 months later showed a shriking mediastinal mass size of 6.9cm × 3.2cm x 7.0cm. He did not demonstrate any shortness of breath on lying flat nor syncopal attacks. Our patient was not planned for mediastinal mass resection in view of his elderly age and it responded to chemoradiotherapy.

## 3 Discussion

Prostate carcinoma is the one of the commonest malignant diseases among males worldwide with an estimated 1,600,000 cases and 366,000 deaths annually [1]. The disease disproportionately affects older adults with the incidence rate rapidly rising as age increases (typically from 50 years old onwards). However, the introduction of PSA as a screening test in the 1980s enabled the detection of prostate carcinoma among younger age groups as well [3]. Diagnostic prostate biopsy is performed either for increased PSA levels above the patient's age cohort or when there is palpable abnormality on digital rectal examination. In the case of our patient, a grossly elevated serum PSA together with positive IHC staining of mediastinal and spine metastasis HPE confirmed our working diagnosis of metastatic prostatic carcinoma. A trucut biopsy was therefore not performed. However, our other possible clinical diagnoses for a widened mediastinum included huge lymphoma, and retrosternal thyroid carcinoma. Although PSA has long been established as a prostatic marker, it is expressed in both benign and malignant prostatic tissues; this thus limits the role of IHC using PSA on tissue sample from needle biopsy [4]. However, if PSA-positive cells are detected in other organs by IHC staining in the context of clinical suspicion of prostate carcinoma, it is likely to represent malignant cells [5].

Studies have reported that most males with prostate carcinoma would die of other causes before the malignancy becomes clinically advanced; it is rare for patients to present with clinical manifestations of prostate carcinoma [6]. At the time of diagnosis, 78% of cases would be localised prostate carcinoma, 12% with reginal lymphadenopathies, and 6% involve distant metastasis [7]. Prostate carcinoma is known to metastasize through lymphatic, hematogenous or direct extension with the pattern of spread largely limited to bones and regional lymph nodes [1,3]. Bone pain therefore may often be the presenting symptom of metastatic prostate carcinoma. Metastasis in other localities is very uncommon; liver, pulmonary, hepatic, retroperitoneal, or abdominal metastasis is rarely the first clinical presentation in such cases. Cetin et al. reported only 1% of mediastinal metastasis originating from prostate carcinoma [8].

Although males with locally advanced disease might be candidates for surgical procedures, for metastatic prostate carcinoma, androgen deprivation therapy (ADT) is the initial approach and could be achieved either by medical castration or with bilateral orchidectomy [9,10]. The rationale for the use of ADT is that prostate carcinoma relies on androgen, with its primary source being the testes, for continued growth. ADT is however associated with significant impairment of quality of life due to its side effects which include sexual dysfunction, gynaecomastia, obesity, and osteoporosis. Nevertheless, meta-analysis and several trials reported no overall survival benefit between immediate and delayed ADT although there was reduction in deaths related to prostate carcinoma [9]. The 5-year relative survival rate for localised and regional prostate carcinoma is 100% compared to 30.6% among metastatic disease [7]. Our patient was commenced on goserelin, a LHRH agonist, with chemoradiotherapy. He responded very well and was able to produce good uninterrupted flow of urine. He did not develop acute urinary retention, which might have required a suprapubic catheter (SPC), or sudden paraplegia. Most importantly, he did not develop stridor or shortness of breath as the mediastinal mass showed reduction in size in response to the ADT and chemoradiotherapy.

Mediastinal masses are undoubtedly anaesthetically challenging. Hemodynamic instability and respiratory compromise would always be an anaesthetic catastrophe due to the compressive effect on the cardiorespiratory systems. Our patient did not exhibit cardiovascular or respiratory compromise and had no preference for any posture. This, however, did not preclude the possibility of an anaesthetic disaster as demonstrated by Yeap et al. [11,12]. We opted for AFOI since this directly enabled us to visualise the airways. In addition, we employed the use of FM ETT with reinforced metal coils to minimise kinking and obstruction. We also avoided usage of muscle relaxants which could cause total airway obstruction from the bulky anterior mediastinal mass. We utilized TCI propofol and remifentanil for maintenance of anaesthesia to assist the spine surgeons in their surgery which was guided by MEP and SSEP. These vital steps led to a safe procedure in a surgery with potentially fatal consequences.

## 4 Conclusion

Patients with advanced prostate carcinoma can present with minimal urinary symptoms. Distant metastasis to the mediastinum is extremely rare. Anaesthesia for patients with a huge mediastinal mass in prone position carries significant cardiorespiratory risks. ADT and chemoradiotherapy can be safely used in patients with metastatic prostatic carcinoma.

## Patient’s perspective

I would like to thank the doctors for their invaluable work in my surgery and subsequent recovery. Their dedication and commitment have allowed me to walk again, for which I am very grateful. I hope my story will inspire our doctors in their noble work and encourage other people with similar conditions to seek medical help because it is never too late to get treatment.

## Ethical approval

Not related as this is a case report.

## Sources of funding

Not available.

## Authors’ contribution

Dr Boon Tat Yeap and Dr Chin Pei Bong were the clinicians and co-authors for this manuscript.

Dr Kai Ming Teah, Dr Kamarudin Mudin and Dr May Zaw Soe were co-authors for this manuscript and assisted in data collection.

## Registration of research studies

This is a case report. No human participants were involved.

Name of the registry:
Unique Identifying number or registration ID:
Hyperlink to your specific registration (must be publicly accessible and will be checked):

## Guarantor

Dr Boon Tat Yeap is the guarantor for this manuscript.

## Consent

Written informed consent was obtained from the patient and parents for publication of this case report and any accompanying images. A copy of the written consent is available for review by the Editor-in-Chief of this journal.

## Provenance and peer review

Not commissioned, externally peer-reviewed.

## Declaration of competing interest

The authors declare that no relevant or material financial interests exist.
